# Perceived social support and symptoms of depression and anxiety in emerging adulthood: A Swedish prospective cohort study

**DOI:** 10.1177/14034948241290927

**Published:** 2024-11-06

**Authors:** Sara Brolin Låftman, Andreas Lundin, Viveca Östberg

**Affiliations:** 1 Department of Public Health Sciences, Stockholm University, Sweden; 2 Centre for Epidemiology and Community Medicine, Region Stockholm, Sweden; 3 Department of Global Public Health, Karolinska Institutet, Sweden

**Keywords:** Social support, adolescence, early adulthood, depression, anxiety, mental health

## Abstract

**Aims:**

The transition from adolescence to young adulthood, often referred to as ‘emerging adulthood’, is a challenging period in life, and mental health problems are common. Although a large number of studies have shown that social support is linked with fewer mental health problems, few longitudinal studies have examined these associations during this life phase. The aim of the current study was to examine the associations between perceived social support from different sources – family, friends and significant other – at age 17–18 and symptoms of depression and anxiety at age 20–21.

**Methods:**

Data were obtained from the cohort study Futura01 based on a Swedish national sample of adolescents attending grade 9 in 2016/2017. We used survey information from 2019 (age 17–18) and 2022 (age 20–21) and linked registry information (*N*=2722). Symptoms of depression and anxiety were measured by the Patient Health Questionnaire-4 (PHQ-4) at age 20–21. Perceived social support was measured by the Multidimensional Scale of Perceived Social Support (MSPSS) at age 17–18. Control variables included sociodemographic characteristics and indicators of mental health problems at age 17–18. Binary logistic regressions were performed.

**Results:**

When mutually adjusting for all sources of perceived social support, family support at age 17–18 had inverse associations with symptoms of both depression and anxiety at age 20–21. Perceived support from friends was associated with subsequent symptoms of anxiety only.

**Conclusions:**

Perceived social support can be a protective factor against mental health problems in emerging adulthood. The family serves a particularly important source of social support.

## Introduction

The transition from adolescence to young adulthood is a life phase characterised by significant development and change. The period from the late teens to the late twenties is often referred to as ‘emerging adulthood’ [1]. During this life phase, individuals often transition from upper secondary school to tertiary education or the labour market, move away from their family of origin and establish romantic relationships. Emerging adulthood is regarded as a critical period due to the high degree of instability and the many challenges faced [1–3], even being defined as ‘the most unstable period of the life span’ [1] (p. 571). Mental health problems are also common during this life phase, with an estimated 12-month prevalence of psychiatric conditions in Sweden ranging from 15% to 20% [4].

Social support is defined as ‘the resources provided by other persons’ [5] (p. 4) and includes different types, such as emotional, instrumental, informational, appraisal and companionship support. According to social support theory, it can enhance health and well-being and act as a protective factor against illness through different processes. The direct effect hypothesis postulates that social support is associated with higher well-being and fewer health problems due to the access to various types of resources through others and the perception that others value and care about you, which, in turn, is linked with positive feelings and an increased sense of self-esteem, stability, security and control. According to the buffering hypothesis, social support can protect against health problems for individuals who are exposed to stressors, either by alleviating or preventing a stress response or by reducing the risk that the stress response translates into adverse health [5,6]. Social support is derived from different sources and can be categorised into primary and secondary groups. Primary groups consist of close, enduring relationships such as family and close friends, which offer concern, care and practical assistance. This everyday support directly benefits health and buffers against stress. Secondary groups, including formal contacts from work and organisations, also play a role. In particular, members of secondary groups who have experienced similar stressors can validate feelings, serve as role models and provide guidance, effectively mitigating stress [7].

A plethora of research has demonstrated that social support is associated with a lower risk of developing psychiatric conditions such as depression and anxiety across the life course [8–11]. However, it has been highlighted that longitudinal studies of the links between social support and mental health outcomes in emerging adulthood are scarce [11,12], despite the fact that social support may be particularly important during this challenging life phase [11]. Yet, some studies exist that show inverse links between support and mental health problems. The majority of these focused on the links between perceived social support and depression [13–15], whereas one recent study covered a broader range of psychiatric conditions [11]. Examining specific sources of social support in this period is also pertinent. Throughout adolescence, individuals experience growing independence from their parents, while peers take on an increasingly significant role. Hence, conducting analyses that examine both parental and peer support in relation to mental health outcomes becomes relevant [15]. These studies should also account for previous mental health issues [11], as mental health may affect levels of perception of support.

In young adulthood, both males and females encounter significant challenges related to education, employment, relocation and forming partnerships [1]. However, young women report higher levels of mental distress and seek treatment for depression and anxiety more frequently than young men do [4]. Additionally, the nature and impact of social support during adolescence can vary by gender. Traditionally, girls are more often socialised to prioritise and value family affiliation and closeness than boys are [16]. In terms of friendships, girls tend to place greater emphasis on psychological aspects such as intimacy and support, whereas boys often prioritise recreational aspects such as companionship and enjoyment [17]. These considerations underscore the importance of examining possible gender differences when investigating the relationship between social support and mental health.

The aim of the present study was to examine the associations between perceived social support from different sources – family, friends and significant other – at age 17–18 and symptoms of depression and anxiety at age 20–21.

## Methods

### Data material and participants

Data were derived from the cohort study Futura01 based on a Swedish national sample of adolescents attending grade 9 in 2016/2017 (the vast majority born in 2001). Statistics Sweden randomly selected 500 schools across Sweden, of which 343 agreed to participate (68.6%). Comparison between participating and non-participating schools did not yield any statistically significant differences in average grades, the proportion of students with highly educated parents or the proportion of students with foreign-born parents [18]. Wave 1 was conducted as a paper-and-pencil survey in classrooms in 2017 when participants attended grade 9 (age 15–16; *N*=5537). Wave 2 was conducted as a web or postal survey in 2019 when participants typically attended the second grade of upper secondary school (age 17–18; *N*=4141). Wave 3 was carried out in 2022 (age 20–21) as a web survey (*N*=3396). A flow chart of the data material is provided in Supplemental Figure S1. The present study was based on data from waves 2 and 3 (*N*=2956 participated in both of these). The attrition between survey waves is addressed in Supplemental Table SI and in the Results and Discussion sections below. The analytical sample includes 2722 participants with valid information on the study variables (92%).

### Outcome variables

Symptoms of depression and anxiety (age 20–21) were measured by the Patient Health Questionnaire-4 (PHQ-4) [19]. This instrument contains the Patient Health Questionnaire-2 (PHQ-2) with two items on symptoms of depression [20] and the Generalized Anxiety Disorder-2 (GAD-2) with two items on symptoms of anxiety [21] over the last two weeks. For both measures, we applied a cut-off score of ⩾3 [19].

### Exposure variables

Perceived social support (age 17–18) was assessed by the 12-item Multidimensional Scale of Perceived Social Support (MSPSS). This instrument was originally created to measure perceived social support in 17- to 22-year-old students in the USA. The MSPSS contains three subscales with four items each, measuring perceived social support from different sources: family, friends and significant other [22]. Example items are ‘My family really tries to help me’ (family subscale), ‘I have friends with whom I can share my joys and sorrows’ (friends subscale) and ‘There is a special person who is around when I am in need’ (significant other subscale). The response categories were based on a seven-point Likert scale, ranging from 1=‘very strongly disagree’ to 7=‘very strongly agree’. For each subscale, we calculated the mean value of the four statements. Internal consistency was high (Cronbach’s alphas were 0.92 for the family subscale, 0.93 for the friends subscale and 0.92 for the significant other subscale). All subscales were highly skewed to the right. Pairwise correlations between the subscales were moderate to strong (family and friends subscales: *r*=0.46, *p*<0.001; family and significant other subscales: *r*=0.56, *p*<0.001; friends and significant other subscales: *r*=0.67, *p*<0.001).

### Covariates

Besides gender, we also included sociodemographic characteristics in terms of parental education, parental country of birth, living arrangements and own education as covariates. Gender was based on information from the participants’ personal security numbers (male or female). Parental education (highest level among parents) and parental country of birth (at least one parent born in Sweden or both parents born in another country) were measured by registry information from 2017. Living arrangements (lives with two parents, lives with one parent, shared residence or other/missing) were measured from self-reported survey information at age 17–18. Own education was measured by upper secondary programme (academic or vocational at age 17–18) derived from self-reported survey information.

Since the PHQ-4 instrument was only introduced in wave 3 (age 20–21), we relied on proxies from wave 2 (age 17–18) to assess prior mental health problems, specifically focusing on medication use and psychosomatic complaints. Medication for depression and anxiety was based on self-reported survey information. Psychosomatic complaints were calculated as a summation index based on items measuring the frequency of headache, stomach ache and difficulties falling asleep [23].

### Statistical method

Binary logistic regression analyses were performed to examine the associations between perceived social support and subsequent symptoms of depression and anxiety. First, we performed crude analyses, including one variable at a time, controlling for gender. Model 1 mutually adjusted for all sources of social support and gender, model 2 added all sociodemographic characteristics and model 3 added indicators of prior mental health problems. To check for multicollinearity, we performed variance inflation factor (VIF) tests, revealing no significant multicollinearity issues among the independent variables (highest VIF=2.25). We also tested for multiplicative interactions between the different sources of perceived social support and between each source of perceived social support and gender. Gender-stratified analyses are presented in Supplemental Tables SII–SV. To take the nested data structure into account, with students clustered in classes in wave 1, robust standard errors were estimated. Stata v18 (StataCorp, College Station, TX) was used for all analyses.

## Results

Descriptives of the analytical sample, consisting of those participating in both waves 2 and 3, are presented in Table I. It is shown that 25.4% reported symptoms of depression and 26.8% reported symptoms of anxiety in wave 3. Analyses of attrition across survey waves, presented in Supplemental Table SI, reveal some systematic bias. Among participants in the wave 1 sample, females and those with parents who had a tertiary education were less likely to drop out in wave 2, whereas those with foreign-born parents were more likely to do so. For those who participated in both waves 1 and 2, individuals reporting high family support in wave 2 had a higher probability of dropping out in wave 3. Additionally, females, those with parents who had a tertiary education and those enrolled in an academic programme in wave 2 were less likely to drop out in wave 3. Conversely, participants living with one parent, those in ‘other’ living arrangements or with missing information in wave 2 were more likely to drop out in wave 3. Notably, medication use and psychosomatic complaints in wave 2 did not predict dropout in wave 3.

**Table I.** Descriptives of the analytical sample (*N*=2722).

	*n*	%		
Symptoms of depression (age 20–21)	690	25.4		
Symptoms of anxiety (age 20–21)	729	26.8		
Gender				
Males (ref.)	1148	42.2		
Females	1574	57.8		
Parental education				
⩽2 years secondary or less	393	14.4		
⩾3 years secondary	519	19.1		
Tertiary	1810	66.5		
Parental country of birth				
At least one in Sweden	2313	85.0		
Two parents outside Sweden	409	15.0		
Living arrangements (age 17–18)				
Lives with two parents	1758	64.6		
Lives with one parent	403	14.8		
Shared residence	310	11.4		
Other/missing	251	9.2		
Upper secondary programme (age 17–18)				
Vocational	513	18.9		
Academic	2129	78.2		
Other programme/other activity/missing	80	2.9		
Medication for depression (age 17–18)	133	4.9		
Medication for anxiety (age 17–18)	139	5.1		
	*M*	*SD*	Min	Max
Psychosomatic complaints (age 17–18)	7.22	2.71	3	15
Perceived social support (MSPSS) (age 17–18)				
Family	5.65	1.39	1	7
Friends	5.61	1.37	1	7
Significant other	6.06	1.26	1	7

*SD*: standard deviation; MSPSS: Multidimensional Scale of Perceived Social Support.

Table II presents results from analyses of perceived social support at age 17–18 and symptoms of depression at age 20–21. The crude analyses show that higher levels of all sources of perceived social support are associated with a lower likelihood of subsequent symptoms of depression. In model 1, mutually adjusting for all three sources of perceived social support and gender, all estimates are attenuated but remain statistically significant. Adding other sociodemographic characteristics in model 2 does not change the results substantially, although the estimate of support from significant other becomes non-significant. When indicators of prior mental health problems are added in model 3, only perceived family support has a statistically significant association with symptoms of depression (odds ratio (OR)=0.89, 95% confidence interval (CI) 0.83–0.96). The estimates of the control variables show that having two parents born abroad, taking medication for depression and reporting psychosomatic complaints at age 17–18 are associated with an increased likelihood of reporting symptoms of depression at age 20–21. Tests for interactions between sources of perceived social support and between each source of perceived social support and gender were not statistically significant (not presented in the table). Gender-stratified analyses (presented in Supplemental Tables SII and SIII) show largely similar patterns among males and females. However, in the fully adjusted models, the estimate for family support is statistically significant in females only.

**Table II.** Associations between perceived social support at age 17–18 and symptoms of depression (PHQ-2) at age 20–21 (*N*=2722).

	Crude^a^		Model 1^b^		Model 2^c^		Model 3^d^	
	OR	95% CI	OR	95% CI	OR	95% CI	OR	95% CI
Perceived social support (MSPSS) (age 17–18)								
Family	0.77***	0.72–0.82	0.84***	0.78–0.90	0.86***	0.79–0.92	0.89**	0.83–0.96
Friends	0.79***	0.74–0.84	0.91*	0.84–0.98	0.91*	0.84–0.99	0.94	0.87–1.02
Significant other	0.76***	0.71–0.81	0.91*	0.83–1.00	0.91	0.83–1.00	0.91	0.83–1.01
Gender								
Males (ref.)	1.00	–	1.00	–	1.00	–	1.00	–
Females	1.18	0.98–1.43	1.29*	1.06–1.57	1.27*	1.05–1.55	1.01	0.82–1.24
Parental education								
⩽2 years secondary or less	1.49**	1.11–2.00			1.34	0.99–1.83	1.36	1.00–1.86
⩾3 years secondary (ref.)	1.00	–			1.00	–	1.00	–
Tertiary	0.89	0.71–1.11			0.99	0.78–1.24	1.03	0.82–1.31
Parental country of birth								
At least one in Sweden (ref.)	1.00	–			1.00	–	1.00	–
Two parents outside Sweden	1.92***	1.52–2.43			1.64***	1.30–2.08	1.70***	1.34–2.16
Living arrangements (age 17–18)								
Lives with two parents (ref.)	1.00	–			1.00	–	1.00	–
Lives with one parent	1.28*	1.01–1.63			1.05	0.82–1.35	0.98	0.76–1.26
Shared residence	1.01	0.77–1.32			1.08	0.82–1.41	1.02	0.78–1.32
Other/missing	1.39*	1.03–1.89			1.23	0.90–1.67	1.16	0.84–1.60
Upper secondary programme (age 17–18)								
Vocational (ref.)	1.00	–			1.00	–	1.00	–
Academic	0.78*	0.62–0.99			0.85	0.66–1.08	0.88	0.69–1.12
Other programme/other activity/missing	1.99*	1.17–3.38			1.79*	1.01–3.17	1.66	0.91–3.01
Medication for depression (age 17–18)	2.85***	1.95–4.16					1.90*	1.12–3.25
Medication for anxiety (age 17–18)	2.14***	1.47–3.14					1.06	0.63–1.80
Psychosomatic complaints (age 17–18)	1.18***	1.14–1.22					1.13***	1.09–1.17

ORs and 95% CIs from binary logistic regressions.

*** *p*<0.001; **p<0.01; *p<0.05.

a Includes one variable at a time, controlling for gender.

b Includes all sources of perceived social support, controlling for gender.

c Includes all sources of perceived social support, controlling for gender and other sociodemographic characteristics.

d Includes all sources of perceived social support, controlling for gender and other sociodemographic characteristics, and indicators of prior mental health problems.

OR: odds ratio; 95% CI: 95% confidence interval; MSPSS: Multidimensional Scale of Perceived Social Support.

Results from analyses of perceived social support at age 17–18 and symptoms of anxiety at age 20–21 are shown in Table III. The crude analyses show statistically significant, inverse associations between all sources of perceived social support and subsequent symptoms of anxiety. In Model 1, including all sources of perceived social support and gender, the associations between support from family and friends and subsequent symptoms of anxiety remain statistically significant. The odds for support from significant other, however, turn positive and non-significant. Adjusting for other sociodemographic characteristics in model 2 does not change the results. Adding indicators of prior mental health problems in model 3 slightly attenuate the estimates of family and friends support, but the overall pattern remains, showing statistically significant associations between perceived support from family (OR=0.81, 95% CI 0.75–0.86) and friends (OR=0.89, 95% CI 0.82–0.98) and later symptoms of anxiety. Furthermore, female gender, attending an academic programme at age 17–18, taking medication for anxiety and reporting psychosomatic complaints at age 17–18 are associated with an increased likelihood of reporting symptoms of anxiety at age 20–21. There were no statistically significant interactions between sources of perceived social support or between sources of perceived social support and gender (not presented in the table). Gender-stratified analyses (presented in Supplemental Tables SIV and SV) demonstrate statistically significant associations between perceived family support and symptoms of anxiety in both genders. The estimates of perceived support from friends are similar for males and females but do not reach statistical significance in the gender separate analyses.

**Table III.** Associations between perceived social support at age 17–18 and symptoms of anxiety (GAD-2) at age 20–21 (*N*=2722).

	Crude^a^		Model 1^b^		Model 2^c^		Model 2^d^	
	OR	95% CI	OR	95% CI	OR	95% CI	OR	95% CI
Perceived social support (MSPSS) (age 17–18)								
Family	0.75***	0.70–0.79	0.76***	0.71–0.82	0.77***	0.72–0.82	0.81***	0.75–0.86
Friends	0.80***	0.75–0.85	0.86***	0.79–0.94	0.86***	0.79–0.93	0.89*	0.82–0.98
Significant other	0.82***	0.76–0.88	1.09	0.99–1.20	1.10	0.99–1.21	1.10	0.99–1.21
Gender								
Males (ref.)	1.00	–	1.00	–	1.00	–	1.00	–
Females	2.49***	2.05–3.02	2.55***	2.08–3.12	2.50***	2.04–3.06	1.86***	1.49–2.31
Parental education								
⩽2 years secondary or less	1.16	0.87–1.56			1.11	0.82–1.51	1.12	0.82–1.52
⩾3 years secondary (ref.)	1.00	–			1.00	–	1.00	–
Tertiary	0.96	0.77–1.20			0.99	0.78–1.25	1.04	0.82–1.33
Parental country of birth								
At least one in Sweden (ref.)	1.00	–			1.00	–	1.00	–
Two parents outside Sweden	1.34*	1.05–1.71			1.19	0.92–1.53	1.24	0.96–1.60
Living arrangements (age 17–18)								
Lives with two parents (ref.)	1.00	–			1.00	–	1.00	–
Lives with one parent	1.40**	1.10–1.78			1.19	0.93–1.53	1.08	0.84–1.39
Shared residence	1.11	0.85–1.47			1.12	0.85–1.49	1.05	0.79–1.39
Other/missing	1.29	0.97–1.72			1.16	0.87–1.54	1.06	0.79–1.42
Upper secondary programme (age 17–18)								
Vocational (ref.)	1.00	–			1.00	–	1.00	–
Academic	1.19	0.95–1.49			1.34*	1.05–1.70	1.44**	1.13–1.83
Other programme/other activity/missing	1.53	0.88–2.65			1.37	0.76–2.47	1.26	0.68–2.36
Medication for depression (age 17–18)	2.77***	1.92–4.01					1.28	0.79–2.07
Medication for anxiety (age 17–18)	3.02***	2.04–4.46					1.88*	1.14–3.10
Psychosomatic complaints (age 17–18)	1.22***	1.18–1.26					1.17***	1.13–1.22

ORs and 95% CIs from binary logistic regressions.

*** *p*<0.001; **p<0.01; *p<0.05.

a Includes one variable at a time, controlling for gender.

b Includes all sources of perceived social support, controlling for gender.

c Includes all sources of perceived social support, controlling for gender and other sociodemographic characteristics.

d Includes all sources of perceived social support, controlling for gender and other sociodemographic characteristics, and indicators of prior mental health problems.

OR: odds ratio; 95% CI: 95% confidence interval; MSPSS: Multidimensional Scale of Perceived Social Support; GAD-2: Generalized Anxiety Disorder-2.

## Discussion

The current study examined the associations between perceived social support from different sources (family, friends and significant other) at age 17–18 and symptoms of depression and anxiety at age 20–21. In the crude analyses, all sources of perceived social support showed inverse, statistically significant associations with symptoms of depression and anxiety alike. However, when mutually adjusting for all sources of support and covariates, only family support showed an inverse association with subsequent symptoms of depression, whereas support from both family and friends was associated with subsequent symptoms of anxiety.

Our findings align with those of previous research, demonstrating inverse links between perceived social support and psychiatric conditions across the life span [8–10], including depression [11,13–15] and anxiety [11] in emerging adulthood. In the present study, perceived social support from the family was identified as particularly important. This finding reflects prior research, which indicated that parental support is more significant than peer support in protecting against depression in children and adolescents [8], and it shows that the central role of the family remains in emerging adulthood. The current study’s finding that perceived support from friends was inversely associated with subsequent symptoms of anxiety in the pooled sample should, however, also be highlighted. A recent study showed that perceived social support from friends during adolescence proved to be especially important for positive mental health outcomes in young adulthood [12]. Our study further indicates that friends can play a protective role in relation to mental health issues such as anxiety, although the association between support from friends and symptoms of anxiety was somewhat weaker compared to the association with family support.

One possible explanation for the greater importance of family support is that families are typically considered primary groups, whereas friendships can exist across both primary and secondary groups, depending on their intimacy, and that support from primary groups is more fundamental to mental health. Additionally, family relationships tend to be more enduring and stable compared to friendships, which are generally more susceptible to change, especially during transitions such as those in education. In our study, most participants graduated from upper secondary school in 2020, spanning the period between the assessments of social support in 2019 and mental health outcomes in 2022. During wave 3, participants were asked, ‘Do you still hang out with the same friends you had in upper secondary school?’ using a seven-point Likert scale ranging from 1=‘no, not at all’ to 7=‘yes, to a very large extent’. Analysis of the responses (not presented) showed that 35.5% of participants in our study sample selected the lowest three values (1–3), 13.9% chose the midpoint value 4 and 50.6% chose the highest three values (5–7). These findings indicate that for a significant number of participants, their friendships had changed over time. Therefore, it appears reasonable that the relatively weaker impact of friend support on mental health outcomes during this period could be partially attributed to these shifts or changes in friendships.

The finding that perceived social support from significant others was not associated with any of the outcomes in the fully adjusted models may be due to the high correlation with support from the other sources, indicating that significant others are often parents, siblings or friends. Our results differ from those of a Finnish study, which observed a direct effect of perceived support from significant others at age 15 on depression at age 20 for girls but no direct effects of support from family or friends [13]. However, the ‘significant other’ subscale is subject to both conceptual and measurement concerns, with suggestions that it might indeed encompass support from both family and friends [24]. The interpretation of the term ‘special person’ may also vary between settings [25].

While we examined the associations between perceived social support and mental health outcomes, it is possible that the results reflect mechanisms pertaining to both the direct effect and the buffering hypothesis. The effects of perceived social support on symptoms of depression and anxiety may partially be attributable to factors such as increased levels of self-esteem and a sense of stability, security and control, as postulated by the direct effect hypothesis. These mechanisms may play a role also in buffering processes [7], wherein potentially stressful situations are less likely to induce stress or lead to mental health conditions [5,6]. Given the numerous challenges that individuals face in emerging adulthood [1–3], it seems likely that stress-buffering mechanisms may also be at work besides a direct effect.

We did not find any statistically significant gender differences in the associations between perceived social support and symptoms of depression and anxiety, as indicated by the non-significant interaction terms and similar patterns in the gender-stratified analyses. However, in the fully adjusted gender-stratified models, the association between family support and symptoms of depression reached statistical significance only among women. Nevertheless, when considered together, the results suggest that perceived social support from family and friends holds largely comparable importance for both females and males. This finding contrasts with the assumption that females, influenced by socialisation processes, place more emphasis on family relationships than males do [16]. It also indicates that while boys and girls may prioritise different aspects of friendship during adolescence [17], the effects of the support of friends on mental health outcomes do not vary by gender. The absence of a statistically significant gender difference in the association between social support and symptoms of depression and anxiety in the current study could be interpreted through the lens of the gender similarities hypothesis [26], supporting the notion that males and females often exhibit more similarities than differences. With regards to the outcomes, the analyses showed that females were more likely to report symptoms of anxiety. Females also had higher odds of reporting symptoms of depression compared with males, although this association was not statistically significant in all models (see also the study by Grigorian et al. [27], which was based on the same data material as the current study). This mirrors the well-documented finding that both depression and anxiety are more common in women than in men [28].

The key strength of the study is the prospective data material with validated measures of both the exposure and the outcome variables, which were measured three years apart. Another merit is the use of the MSPSS, as prospective studies based on validated measures of social support have been called for [8]. However, one limitation is the non-response in several stages. In the baseline survey, there was attrition at both the school and the student level. Although several school-related characteristics did not differ between participating and non-participating schools [18], it is possible that the attrition at the student level was systematic. The baseline survey was conducted in school, and students who were absent on the day of the survey were probably likely to have more health problems on average. There has also been systematic attrition across survey waves by sociodemographic characteristics. While there was no selective attrition with regards to perceived social support from friends or significant others or health-related variables between waves 2 and 3, participants with higher levels of family support in wave 2 had an increased probability of dropping out in wave 3. However, it is more likely that this attrition bias may have implied an underestimation of the association between family support and mental health outcomes rather than the other way around. Furthermore, one weakness is that we were unable to control for self-reported symptoms of depression and anxiety at age 17–18. To account for prior mental health problems, we included self-reported information on medication for depression and anxiety and psychosomatic complaints from this time point as proxies. It should be underscored, however, that our measures of medication use only capture participants who had received this specific type of treatment. Additionally, our measure of psychosomatic complaints was limited to three items with a strong somatic focus (whereas other measures typically include multiple items that address both psychological and somatic components; e.g., Högberg et al. [29]). Due to these limitations, we may fail to capture all individuals with symptoms of depression and anxiety at the age of 17–18. Thus, we cannot fully determine the temporal order of the associations between perceived social support and symptoms of depression and anxiety, as individuals with depression and anxiety may be less inclined to seek social support and/or may perceive their possibilities of getting social support in a more negative light. It is also possible that a low level of sociability is associated with both low support and mental health problems [13]. Another limitation concerns the fact that in our measure of family support, we could not distinguish between maternal and paternal support, which may have differing effects on mental health outcomes [8,12], as well as in relation to the gender of the child [14]. Furthermore, given that a significant proportion of adolescents in Sweden have experienced parental separation or divorce (specifically, 28% by the age of 15 [30]), it is crucial to distinguish between the effects of material and paternal support while also considering the diversity of family types. It is important to note, however, that in Sweden, parental separation or divorce does not automatically mean that a child maintains close contact with only one parent, as shared residence is a common post-separation arrangement [31]. Finally, the MSPSS instrument does not distinguish between various types of social support, limiting our ability to draw conclusions about their specific effects. Future studies should investigate support from both family and friends, considering different types of social support from these sources – such as emotional, instrumental, informational, appraisal and companionship support – and their links to mental health. While the present study was limited to a three-year time span during emerging adulthood, another relevant endeavour for future research is to track adolescents into mid- and late adulthood in order to examine the potential long-term effects of perceived social support during adolescence on mental health later in life, as well as to study the underlying mechanisms. For instance, it can be assumed that perceived social support during adolescence equips individuals with the abilities and confidence needed to establish healthy relationships with peers and partners in adulthood, potentially contributing to improved mental health [15].

In conclusion, the findings of this study indicate that perceived social support in late adolescence can act as a protective factor against mental health problems in young adulthood, with the family serving as a particularly significant source of social support. Therefore, awareness about the importance of family support also in late adolescence and interventions that can improve the family climate in this age group may be relevant [32].

## Supplemental Material

sj-docx-1-sjp-10.1177_14034948241290927 – Supplemental material for Perceived social support and symptoms of depression and anxiety in emerging adulthood: A Swedish prospective cohort study Supplemental material, sj-docx-1-sjp-10.1177_14034948241290927 for Perceived social support and symptoms of depression and anxiety in emerging adulthood: A Swedish prospective cohort study by Sara Brolin Låftman, Andreas Lundin and Viveca Östberg in Scandinavian Journal of Public Health
