# Supplementary material for: Perceived social support and symptoms of depression and anxiety in emerging adulthood: A Swedish prospective cohort study
Source: Scand J Public Health. 2024 Nov 6;53(8):854–62. doi: 10.1177/14034948241290927 (PMC12619846; doi:10.1177/14034948241290927)
Supplement: sj-docx-1-sjp-10.1177_14034948241290927 – Supplemental material for Perceived social support and symptoms of depression and anxiety in emerging adulthood: A Swedish prospective cohort study [file sj-docx-1-sjp-10.1177_14034948241290927.docx]

School sample, wave 1, 2017:

n_schools_=500

n_students_=2,956^d^

Schools that agreed

to participate, wave 1, 2017:

nschools=343 n_students_=6,769^a^

Excluded due to incorrect/unreadable personal security numbers, non-response on central questions, unreliable responses, or retracted consent: n_students_=185

Students that agreed to participate, wave 1, 2017:

n_students_=5,722^b^

Supplementary Material. Fig. S1. Flow chart of Futura01, waves 1-3.

^a^ Present at school on the day of the classroom survey.

^b^ Responded to the classroom survey.

^c^ Responded to the web survey (83%) or the postal survey (17%).

^d^ Participated in all three surveys: waves 1-3. Study population of the present study, on which the analytical sample (n=2,722) was based.

^e^ Participated in waves 1 and 3, but not in wave 2.

Study population, age 20-21, wave 3, 2022:

n_students_=3,396

Study population, age 17-18, wave 2, 2019:

n_students_=4,141^c^

n_students_=440^e^

Study population, age 15-16, wave 1, 2017:

n_students_=5,537

Supplementary Material. Table S1. Dropout in wave 2 (age 17-18) by study variables from wave 1 (age 15-16) among those participating in wave 1 (upper part of the table); and dropout in wave 3 (age 20-21) by study variables from wave 1 and wave 2 among those participating in wave 2 (lower part of the table). Coefficients from linear probability models (b) and 95% confidence intervals (CI) from fully adjusted regression models.

|  | Dropout in wave 2  (n=5,340)^a^ | |
| --- | --- | --- |
| Variables from wave 1 | b | 95% CI |
| Gender |  |  |
| Males (ref.) | 0.00 | - |
| Females | -0.12*** | -0.14, -0.10 |
| Parental education |  |  |
| ≤2 years secondary or less | 0.04 | 0.00, 0.07 |
| ≥3 years secondary (ref.) | 0.00 | - |
| Tertiary | -0.08*** | -0.11, -0.06 |
| Parental country of birth |  |  |
| At least one in Sweden (ref.) | 0.00 | - |
| Two parents outside Sweden | 0.07*** | 0.04, 0.11 |
|  |  |  |
|  | Dropout in wave 3  (n=3,797)^b^ | |
| Variables from wave 1 and 2 | b | 95% CI |
| Perceived social support (MSPSS) (age 17-18) |  |  |
| Family | 0.02** | 0.00, 0.03 |
| Friends | 0.00 | -0.01, 0.02 |
| Significant other | 0.00 | -0.01, 0.01 |
| Gender |  |  |
| Males (ref.) | 0.00 | - |
| Females | -0.09*** | -0.12, -0.06 |
| Parental education |  |  |
| ≤2 years secondary or less | 0.00 | -0.05, 0.05 |
| ≥3 years secondary (ref.) | 0.00 | - |
| Tertiary | -0.05** | -0.09, -0.02 |
| Parental country of birth |  |  |
| At least one in Sweden (ref.) | 0.00 | - |
| Two parents outside Sweden | 0.02 | -0.01, 0.06 |
| Living arrangements (age 17-18) |  |  |
| Lives with two parents (ref.) | 0.00 | - |
| Lives with one parent | 0.05** | 0.01, 0.09 |
| Shared residence | 0.01 | -0.03, 0.06 |
| Other/missing | 0.05* | 0.00, 0.10 |
| Upper secondary programme (age 17-18) |  |  |
| Vocational (ref.) | 0.00 | - |
| Academic | -0.09*** | -0.13, -0.05 |
| Other programme/other activity/missing | 0.05 | -0.04, 0.13 |
| Medication for depression (age 17-18) | -0.02 | -0.10, 0.07 |
| Medication for anxiety (age 17-18) | 0.03 | -0.05, 0.12 |
| Psychosomatic complaints (age 17-18) | 0.01 | 0.00, 0.01 |

*** p<0.001 **p<0.01 *p<0.05

^a^ Analysis based on cases with complete information on all study variables from wave 1.

^b^ Analysis based on cases with complete information on all study variables from waves 1 and 2.

Supplementary Material. Table S2. Associations between perceived social support at age 17-18 and depression symptoms (PHQ-2) at age 20-21 in males. Odds ratios (OR) and 95% confidence intervals (95% CI) from binary logistic regressions. n=1,148

|  | Crude^a^ | | Model 1^b^ | | Model 2^c^ | | Model 3^d^ | |
| --- | --- | --- | --- | --- | --- | --- | --- | --- |
|  | OR | 95% CI | OR | 95% CI | OR | 95% CI | OR | 95% CI |
| Perceived social support (MSPSS) (age 17-18) |  |  |  |  |  |  |  |  |
| Family | 0.76*** | 0.68-0.84 | 0.84** | 0.74-0.96 | 0.86* | 0.76-0.98 | 0.93 | 0.81-1.07 |
| Friends | 0.78*** | 0.72-0.86 | 0.90 | 0.80-1.02 | 0.91 | 0.80-1.03 | 0.92 | 0.81-1.05 |
| Significant other | 0.78*** | 0.72-0.86 | 0.93 | 0.81-1.06 | 0.92 | 0.81-1.06 | 0.92 | 0.80-1.05 |
| Parental education |  |  |  |  |  |  |  |  |
| ≤2 years secondary or less | 1.45 | 0.89-2.36 |  |  | 1.30 | 0.78-2.18 | 1.38 | 0.81-2.35 |
| ≥3 years secondary (ref.) | 1.00 | - |  |  | 1.00 | - | 1.00 | - |
| Tertiary | 0.95 | 0.65-1.37 |  |  | 1.07 | 0.73-1.57 | 1.15 | 0.77-1.70 |
| Parental country of birth |  |  |  |  |  |  |  |  |
| At least one in Sweden (ref.) | 1.00 | - |  |  | 1.00 | - | 1.00 | - |
| Two parents outside Sweden | 2.47*** | 1.71-3.56 |  |  | 2.25*** | 1.56-3.26 | 2.28*** | 1.57-3.31 |
| Living arrangements (age 17-18) |  |  |  |  |  |  |  |  |
| Lives with two parents (ref.) | 1.00 | - |  |  | 1.00 | - | 1.00 | - |
| Lives with one parent | 1.47 | 1.00-2.16 |  |  | 1.23 | 0.80-1.88 | 1.12 | 0.73-1.72 |
| Shared residence | 1.16 | 0.74-1.81 |  |  | 1.37 | 0.88-2.15 | 1.32 | 0.85-2.05 |
| Other/missing | 1.11 | 0.67-1.83 |  |  | 0.99 | 0.58-1.69 | 0.98 | 0.56-1.71 |
| Upper secondary programme (age 17-18) |  |  |  |  |  |  |  |  |
| Vocational (ref.) | 1.00 | - |  |  | 1.00 | - | 1.00 | - |
| Academic | 0.85 | 0.60-1.19 |  |  | 0.84 | 0.58-1.21 | 0.89 | 0.61-1.28 |
| Other programme/other activity/missing | 2.61* | 1.26-5.40 |  |  | 2.38* | 1.02-5.54 | 2.39* | 1.01-5.68 |
| Medication for depression (age 17-18) | 2.70** | 1.39-5.24 |  |  |  |  | 1.14 | 0.46-2.84 |
| Medication for anxiety (age 17-18) | 2.44* | 1.15-5.20 |  |  |  |  | 1.65 | 0.64-4.22 |
| Psychosomatic complaints (age 17-18) | 1.21*** | 1.15-1.28 |  |  |  |  | 1.17*** | 1.10-1.24 |

*** p<0.001 **p<0.01 *p<0.05

^a^ Includes one variable at a time.

^b^ Includes all sources of perceived social support.

^c^ Includes all sources of perceived social support, controlling for sociodemographic characteristics.

^d^ Includes all sources of perceived social support, controlling for sociodemographic characteristics and indicators of prior mental health problems.

Supplementary Material. Table S3. Associations between perceived social support at age 17-18 and depression symptoms (PHQ-2) at age 20-21 in females. Odds ratios (OR) and 95% confidence intervals (95% CI) from binary logistic regressions. n=1,574

|  | Crude^a^ | | Model 1^b^ | | Model 2^c^ | | Model 3^d^ | |
| --- | --- | --- | --- | --- | --- | --- | --- | --- |
|  | OR | 95% CI | OR | 95% CI | OR | 95% CI | OR | 95% CI |
| Perceived social support (MSPSS) (age 17-18) |  |  |  |  |  |  |  |  |
| Family | 0.77*** | 0.72-0.83 | 0.84*** | 0.77-0.92 | 0.85** | 0.78-0.93 | 0.88** | 0.80-0.96 |
| Friends | 0.79*** | 0.73-0.86 | 0.92 | 0.82-1.02 | 0.92 | 0.83-1.03 | 0.96 | 0.86-1.08 |
| Significant other | 0.73*** | 0.66-0.80 | 0.88 | 0.77-1.01 | 0.88 | 0.77-1.02 | 0.88 | 0.76-1.02 |
| Parental education |  |  |  |  |  |  |  |  |
| ≤2 years secondary or less | 1.51* | 1.04-2.19 |  |  | 1.37 | 0.92-2.06 | 1.38 | 0.92-2.07 |
| ≥3 years secondary (ref.) | 1.00 | - |  |  | 1.00 | - | 1.00 | - |
| Tertiary | 0.85 | 0.63-1.14 |  |  | 0.94 | 0.69-1.29 | 0.98 | 0.71-1.35 |
| Parental country of birth |  |  |  |  |  |  |  |  |
| At least one in Sweden (ref.) | 1.00 | - |  |  | 1.00 | - | 1.00 | - |
| Two parents outside Sweden | 1.62** | 1.22-2.16 |  |  | 1.35 | 0.99-1.84 | 1.42* | 1.04-1.94 |
| Living arrangements (age 17-18) |  |  |  |  |  |  |  |  |
| Lives with two parents (ref.) | 1.00 | - |  |  | 1.00 | - | 1.00 | - |
| Lives with one parent | 1.19 | 0.87-1.62 |  |  | 0.97 | 0.71-1.33 | 0.91 | 0.66-1.26 |
| Shared residence | 0.92 | 0.65-1.30 |  |  | 0.93 | 0.65-1.33 | 0.88 | 0.61-1.25 |
| Other/missing | 1.57* | 1.10-2.25 |  |  | 1.37 | 0.95-1.97 | 1.26 | 0.87-1.83 |
| Upper secondary programme (age 17-18) |  |  |  |  |  |  |  |  |
| Vocational (ref.) | 1.00 | - |  |  | 1.00 | - | 1.00 | - |
| Academic | 0.74* | 0.55-0.99 |  |  | 0.86 | 0.63-1.18 | 0.88 | 0.64-1.21 |
| Other programme/other activity/missing | 1.49 | 0.72-3.06 |  |  | 1.40 | 0.67-2.94 | 1.24 | 0.57-2.68 |
| Medication for depression (age 17-18) | 2.91*** | 1.83-4.62 |  |  |  |  | 2.28* | 1.22-4.27 |
| Medication for anxiety (age 17-18) | 2.08** | 1.35-3.20 |  |  |  |  | 0.94 | 0.52-1.72 |
| Psychosomatic complaints (age 17-18) | 1.16*** | 1.11-1.21 |  |  |  |  | 1.11*** | 1.06-1.16 |

*** p<0.001 **p<0.01 *p<0.05

^a^ Includes one variable at a time.

^b^ Includes all sources of perceived social support.

^c^ Includes all sources of perceived social support, controlling for sociodemographic characteristics.

^d^ Includes all sources of perceived social support, controlling for sociodemographic characteristics and indicators of prior mental health problems.

Supplementary Material. Table S4. Associations between perceived social support at age 17-18 and anxiety symptoms (GAD-2) at age 20-21 in males. Odds ratios (OR) and 95% confidence intervals (95% CI) from binary logistic regressions. n=1,148

|  | Crude^a^ | | Model 1^b^ | | Model 2^c^ | | Model 2^d^ | |
| --- | --- | --- | --- | --- | --- | --- | --- | --- |
|  | OR | 95% CI | OR | 95% CI | OR | 95% CI | OR | 95% CI |
| Perceived social support (MSPSS) (age 17-18) |  |  |  |  |  |  |  |  |
| Family | 0.73*** | 0.66-0.81 | 0.72*** | 0.64-0.82 | 0.72*** | 0.63-0.82 | 0.77*** | 0.67-0.88 |
| Friends | 0.81*** | 0.73-0.90 | 0.86* | 0.75-0.99 | 0.88 | 0.76-1.03 | 0.90 | 0.77-1.05 |
| Significant other | 0.86** | 0.78-0.95 | 1.16 | 0.99-1.36 | 1.17 | 1.00-1.38 | 1.17 | 0.99-1.37 |
| Parental education |  |  |  |  |  |  |  |  |
| ≤2 years secondary or less | 1.11 | 0.63-1.94 |  |  | 1.17 | 0.64-2.11 | 1.22 | 0.67-2.23 |
| ≥3 years secondary (ref.) | 1.00 | - |  |  | 1.00 | - | 1.00 | - |
| Tertiary | 0.97 | 0.66-1.42 |  |  | 1.04 | 0.70-1.54 | 1.10 | 0.74-1.64 |
| Parental country of birth |  |  |  |  |  |  |  |  |
| At least one in Sweden (ref.) | 1.00 | - |  |  | 1.00 | - | 1.00 | - |
| Two parents outside Sweden | 2.07** | 1.37-3.12 |  |  | 1.84** | 1.22-2.78 | 1.84** | 1.21-2.80 |
| Living arrangements (age 17-18) |  |  |  |  |  |  |  |  |
| Lives with two parents (ref.) | 1.00 | - |  |  | 1.00 | - | 1.00 | - |
| Lives with one parent | 1.69* | 1.11-2.55 |  |  | 1.41 | 0.91-2.19 | 1.30 | 0.84-2.01 |
| Shared residence | 1.10 | 0.67-1.81 |  |  | 1.22 | 0.73-2.05 | 1.19 | 0.72-1.97 |
| Other/missing | 0.67 | 0.36-1.25 |  |  | 0.55 | 0.30-1.01 | 0.53* | 0.30-0.97 |
| Upper secondary programme (age 17-18) |  |  |  |  |  |  |  |  |
| Vocational (ref.) | 1.00 | - |  |  | 1.00 | - | 1.00 | - |
| Academic | 1.54* | 1.00-2.36 |  |  | 1.62* | 1.01-2.62 | 1.74* | 1.09-2.76 |
| Other programme/other activity/missing | 3.09** | 1.40-6.84 |  |  | 2.97* | 1.22-7.23 | 3.07* | 1.24-7.60 |
| Medication for depression (age 17-18) | 1.91 | 0.91-4.03 |  |  |  |  | 0.93 | 0.41-2.14 |
| Medication for anxiety (age 17-18) | 1.82 | 0.74-4.50 |  |  |  |  | 1.41 | 0.48-4.10 |
| Psychosomatic complaints (age 17-18) | 1.21*** | 1.14-1.28 |  |  |  |  | 1.16*** | 1.09-1.24 |

*** p<0.001 **p<0.01 *p<0.05

^a^ Includes one variable at a time.

^b^ Includes all sources of perceived social support.

^c^ Includes all sources of perceived social support, controlling for sociodemographic characteristics.

^d^ Includes all sources of perceived social support, controlling for sociodemographic characteristics and indicators of prior mental health problems.

Supplementary Material. Table S5. Associations between perceived social support at age 17-18 and anxiety symptoms (GAD-2) at age 20-21 in females. Odds ratios (OR) and 95% confidence intervals (95% CI) from binary logistic regressions. n=1,574

|  | Crude^a^ | | Model 1^b^ | | Model 2^c^ | | Model 2^d^ | |
| --- | --- | --- | --- | --- | --- | --- | --- | --- |
|  | OR | 95% CI | OR | 95% CI | OR | 95% CI | OR | 95% CI |
| Perceived social support (MSPSS) (age 17-18) |  |  |  |  |  |  |  |  |
| Family | 0.75*** | 0.70-0.81 | 0.78*** | 0.72-0.85 | 0.79*** | 0.72-0.86 | 0.82*** | 0.75-0.90 |
| Friends | 0.79*** | 0.73-0.86 | 0.87** | 0.79-0.96 | 0.87** | 0.78-0.96 | 0.91 | 0.82-1.02 |
| Significant other | 0.78*** | 0.70-0.86 | 1.03 | 0.91-1.18 | 1.03 | 0.90-1.17 | 1.03 | 0.90-1.18 |
| Parental education |  |  |  |  |  |  |  |  |
| ≤2 years secondary or less | 1.18 | 0.83-1.68 |  |  | 1.13 | 0.78-1.64 | 1.11 | 0.77-1.61 |
| ≥3 years secondary (ref.) | 1.00 | - |  |  | 1.00 | - | 1.00 | - |
| Tertiary | 0.96 | 0.73-1.26 |  |  | 0.99 | 0.73-1.33 | 1.03 | 0.76-1.40 |
| Parental country of birth |  |  |  |  |  |  |  |  |
| At least one in Sweden (ref.) | 1.00 | - |  |  | 1.00 | - | 1.00 | - |
| Two parents outside Sweden | 1.08 | 0.81-1.43 |  |  | 0.96 | 0.70-1.31 | 1.02 | 0.75-1.41 |
| Living arrangements (age 17-18) |  |  |  |  |  |  |  |  |
| Lives with two parents (ref.) | 1.00 | - |  |  | 1.00 | - | 1.00 | - |
| Lives with one parent | 1.30 | 0.96-1.74 |  |  | 1.11 | 0.81-1.52 | 0.98 | 0.72-1.35 |
| Shared residence | 1.12 | 0.81-1.56 |  |  | 1.11 | 0.79-1.55 | 1.01 | 0.72-1.44 |
| Other/missing | 1.61** | 1.15-2.26 |  |  | 1.45* | 1.02-2.05 | 1.30 | 0.91-1.87 |
| Upper secondary programme (age 17-18) |  |  |  |  |  |  |  |  |
| Vocational (ref.) | 1.00 | - |  |  | 1.00 | - | 1.00 | - |
| Academic | 1.06 | 0.81-1.39 |  |  | 1.26 | 0.94-1.68 | 1.33 | 1.00-1.79 |
| Other programme/other activity/missing | 0.86 | 0.41-1.84 |  |  | 0.80 | 0.37-1.76 | 0.67 | 0.29-1.55 |
| Medication for depression (age 17-18) | 3.14*** | 2.03-4.84 |  |  |  |  | 1.39 | 0.77-2.51 |
| Medication for anxiety (age 17-18) | 3.37*** | 2.20-5.17 |  |  |  |  | 1.98* | 1.13-3.48 |
| Psychosomatic complaints (age 17-18) | 1.23*** | 1.17-1.28 |  |  |  |  | 1.18*** | 1.13-1.24 |

*** p<0.001 **p<0.01 *p<0.05

^a^ Includes one variable at a time.

^b^ Includes all sources of perceived social support.

^c^ Includes all sources of perceived social support, controlling for sociodemographic characteristics.

^d^ Includes all sources of perceived social support, controlling for sociodemographic characteristics and indicators of prior mental health problems.
